# Airway management in critically ill patients

**DOI:** 10.1016/j.bjae.2025.05.003

**Published:** 2025-06-30

**Authors:** V. Russotto, M. Sorbello

**Affiliations:** 1 University Hospital San Luigi Gonzaga, Turin, Italy; 2 Hospital Giovanni Paolo II, Ragusa, Italy

**Keywords:** airway management, critical care, intubation

**Learning objectives**

By reading this article, you should be able to:

• Identify the most important features of airway management in critically ill patients.
• Describe evidence-based strategies to optimise haemodynamics and oxygenation during intubation.
• Explain evidence-based strategies to maximise the success of tracheal intubation first time.

**Key points**

• Airway management in critically ill patients is associated with high rates of major adverse events, with hypotension or need for rescue vasopressors in up to 43% of procedures.
• Patients with moderate-to-severe hypoxaemia and significantly reduced functional residual capacity should receive positive pressure preoxygenation to increase the safe apnoea time.
• The patient should be optimised haemodynamically and i.v. anaesthetic agents selected carefully before tracheal intubation.
• Videolaryngoscopy increases first-pass intubation success rate and should be considered for all critically ill patients needing intubation.

## The physiologically difficult airway

Airway management in the critically ill patient is a challenging task, with an estimated 45% risk of adverse events. Cardiovascular collapse (defined in most studies as a systolic pressure <65 mmHg at least once, or 90 mmHg for >30 min; or the need to start or increase vasopressor drugs or treat with rescue i.v. fluids) is observed in up to 43% of procedures. Severe hypoxaemia (*S*po_2_ <80%) is reported in 9%, and cardiac arrest reported in 3% of procedures.^1^ Failure to achieve first-pass tracheal intubation significantly increases the risks after intubation, and patients who sustain a major adverse event after intubation are at higher risk of both intensive care unit (ICU) (adjusted odds ratio [aOR] 1.52, 95% confidence interval [95% CI] 1.26–1.83); and 28-day mortality (aOR 1.44, 95% CI 1.19–1.74).^1^ The significantly higher frequency of adverse events in critical care, compared with the perioperative setting, results partly from the underlying deranged physiology and poor functional reserve of ICU patients. Underlying systolic dysfunction, diastolic dysfunction, or both, hypoxaemia or acidosis exposes ICU patients to increased harm arising from peri-induction hypotension, apnoea and transition from negative to positive intrathoracic pressure.^2^ These specific challenges have been defined as *physiologically difficult airways* in contrast to the *anatomical airway challenges* which may be more commonly encountered in elective perioperative care.^3^ Nevertheless, anatomical challenges may be encountered in critically ill patients. Suboptimal availability of devices (e.g. videolaryngoscopes or intubation adjuncts such as bougies) and expert operators, together with difficult ergonomics (e.g. the presence of cumbersome monitoring or organ support devices reducing the space around patient’s bed and hampering the operator’s optimal position for airway access), may increase the risks associated with airway management in both the emergency department (ED) and ICU.^1^^,^^3^^,^^4^ In such scenarios, airway assessment is often time-critical and necessarily brief because intubation cannot be postponed. Given the need to maximise first-pass intubation success, focused airway evaluation should be performed in proportion to the time available and addressing the most critical aspects of airway anatomy for airway instrumentation, such as interincisor distance and the upper lip bite test (a reliable predictor of difficult intubation).^5^^,^^6^ The MACOCHA score is one predictive composite airway assessment tool designed for critically ill patients, consisting of: Mallampati score, obstructive sleep Apnoea, Cervical spine movement limitation, reduced mouth Opening, Coma, Hypoxaemia and non-Anaesthetist operator. The MACOCHA score incorporates two crucial factors observed in critically ill patients: level of consciousness (indicating likely presence or absence of airway protective reflexes and underlying respiratory drive) and the operator’s training.^7^ In a validation cohort, the MACOCHA score had an area under the curve for success of 0.86 (95% CI 0.76–0.96), with a sensitivity of 73%, a specificity of 89%, a negative predictive value of 98% and a positive predictive value of 36%.^7^ Its adoption is recommended by international guidelines on airway management in the ICU,^3^^,^^8^ although a survey assessing how commonly this score is implemented in daily practice is lacking.

## Haemodynamic optimisation during intubation

**Fig 1:**
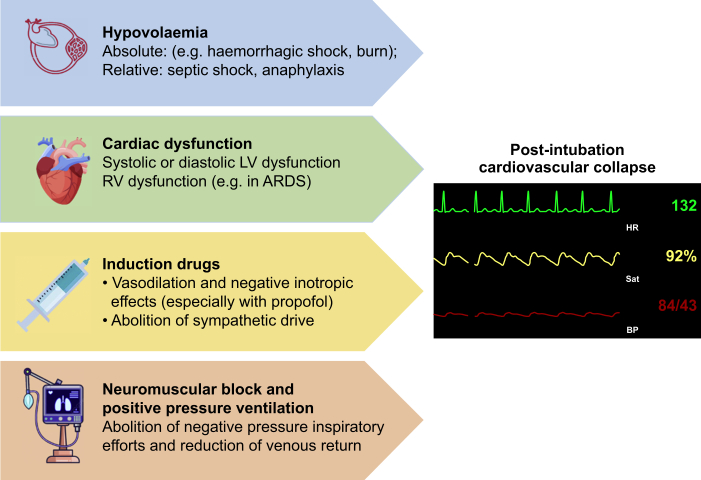
Pathophysiology mechanisms involved in cardiovascular collapse after intubation in critically ill patients.

The high risk of cardiovascular collapse and cardiac arrest during airway management in critically ill patients results from the interplay of patient and airway management strategies.^9^ Figure 1 summarises the main pathophysiology mechanisms of cardiovascular collapse after intubation in critically ill patients. Preexisting absolute (e.g. haemorrhagic shock) or relative (e.g. septic shock) hypovolaemia are common in ICU patients requiring intubation, along with different degrees of systolic or diastolic left ventricular dysfunction.^3^ Patients with shock or ventricular dysfunction tolerate the transition from negative pressure to positive pressure ventilation poorly. Drug-induced vasoplegia and reduced cardiac contractility may exacerbate cardiovascular collapse.^3^^,^^9^ These effects may be particularly severe in older, more frail patients.^10^ Finally, patients with acute respiratory distress syndrome (ARDS), or other respiratory disorders necessitating ICU admission, may have reduced functional residual capacity (FRC) and increased pulmonary vascular resistance, which may impair right ventricular function at the time of induction of anaesthesia for airway management.^3^

In all critically ill patients needing emergency intubation, a focused assessment of the haemodynamic status must be performed, and volume optimisation, preemptive treatment with vasopressors, or both to mitigate cardiovascular collapse after intubation.^3^^,^^9^

Two important trials in this area are the PrePARE and PrePARE II studies. The PrePARE trial randomised critically ill patients undergoing emergency intubation to either a 500 ml i.v. crystalloid bolus before induction or to no fluid bolus, for the prevention of post-intubation cardiovascular collapse.^11^ PrePARE II replicated this randomisation for selected patients receiving positive pressure preoxygenation.^12^ Although neither trial showed benefit of fluid loading for prevention of cardiovascular collapse it should be emphasised that the trial interventions were not treatment of hypovolaemia, which we consider pivotal and which must follow a personalised approach, but rather the mandatory treatment with i.v. fluids in unselected critically ill patients.

Critically ill patients usually have an activated adrenergic drive (often further enhanced by agitation and pain) and observable as tachycardia with a normal or even increased arterial pressure. Blunting of the compensatory sympathetic pathophysiology response by hypnotic or analgesic medications may contribute to cardiovascular collapse observed after tracheal intubation. This, and the differing vasodilatory effects of hypnotic medications, is the rationale behind the pre-emptive treatment with vasoactive medications currently under investigation in the international multicentre randomised trial PREVENTION (NCT05014581).

## Strategies to optimise oxygenation

**Fig 2:**
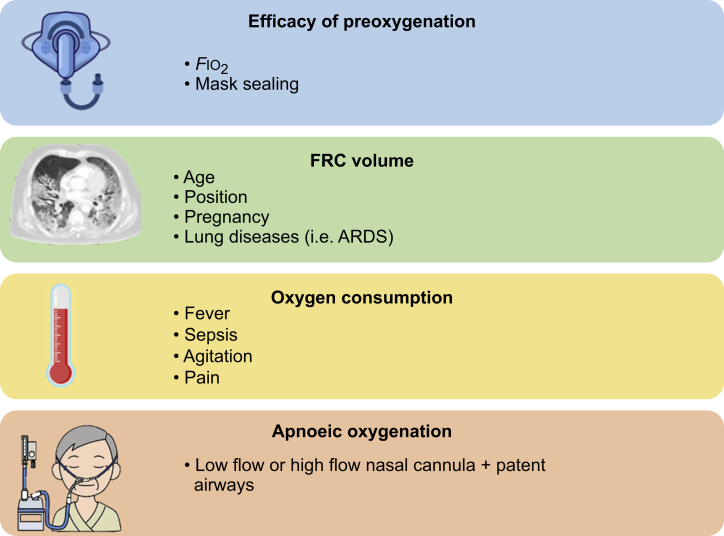
Key variables associated with effective preoxygenation and optimisation of the safe apnoea period.

Severe hypoxaemia (*S*po_2_ <80%) is the second most common adverse event complicating emergency tracheal intubation in critically ill patients, occurring in 9.3% of procedures.^1^ The safe apnoea time is the time for haemoglobin saturation to decrease from 100% to 90%, which represents a critical level beyond which rapid profound desaturation occurs.^13^ Extension of the safe apnoea time during airway instrumentation is of paramount importance to minimise hypoxic complications. Storage of oxygen is very difficult in a biological system. In a healthy adult breathing 100% oxygen, the largest increase of oxygen stores occurs in the FRC. Functional residual capacity is determined partly by the patient’s age, height, and, importantly, body position. Functional residual capacity in healthy individuals in the sitting position is ∼30–35 ml kg^−1^. However, in critically ill patients, FRC can be profoundly reduced. As an example, ARDS is associated with a marked reduction of aerated lung (e.g. in a patient with a respiratory system compliance of 40 cmH_2_O ml^−1^, ventilated lung may be only 40% of total lung capacity).^14^ Consequently FRC, and therefore safe apnoea time, are reduced. During apnoea in a paralysed healthy individual, total body oxygen consumption ($\dot{V}$o_2_) is approximately constant at 230 ml min^−1^. Conditions such as sepsis, fever, agitation and pain significantly increase oxygen consumption. Figure 2 illustrates the key variables associated with effective preoxygenation and maximisation of the safe apnoea time.

Multiple preoxygenation strategies are described, with different patient-device interfaces and durations, but most share the common target of an *F*io_2_ of 100%.^13^ A reduction in FRC hampers the effectiveness of conventional preoxygenation strategies in critically ill patients. Baillard and colleagues randomised hypoxaemic patients requiring tracheal intubation to preoxygenation via non-invasive pressure-support ventilation with positive end-expiratory pressure (PEEP), or to preoxygenation via a standard non-rebreathing bag-mask, testing the effect on desaturation and hypoxaemia during and after tracheal intubation.^15^ Patients randomised to non-invasive pressure-support ventilation with PEEP had significantly increased *S*po_2_ and *P*ao_2_ values at the end of preoxygenation and at 5 and 30 min after intubation. The observed benefit may be explained by the increase of FRC brought about by pressure-support ventilation with accompanying prolongation of the safe apnoea time.^15^ In the FLORALI-2 trial, 313 adults with a *P*ao_2_/*F*io_2_ ratio of ≤300 mmHg needing intubation were randomised to high-flow nasal oxygen (HFNO) or non-invasive ventilation (NIV) for preoxygenation.^16^ While the occurrence of severe hypoxaemia was not different between groups in the overall study population, subgroup analysis of those patients with moderate or severe hypoxaemia (*P*ao_2_/*F*io_2_ ≤200 mmHg) indicated less frequent severe hypoxaemia after preoxygenation with NIV compared with HFNO.^16^ However, it should be highlighted that the benefit of NIV over HFNO has been described in a subgroup analysis, and further studies are needed to confirm this finding in patients with moderate-to-severe hypoxaemia.^16^

Most recently, the PREOXI trial confirmed the benefit of NIV over traditional facemask oxygenation to reduce hypoxaemia in critically ill patients undergoing intubation.^17^ Notably, the control group of the PREOXI trial was represented by either a non-rebreathing mask or a bag-mask without manual ventilation, and this should be considered for the interpretation of results and their general application.^17^

Apnoeic oxygenation, first described by Franz Volhard in 1908, is based on the principle of a pressure gradient between the upper airway and alveoli and subsequent mass movement of oxygen down the trachea into the alveoli, driven by the reduction of gas volume from oxygen absorption at a net exchange rate of 210 ml min^−1^ (equal to a $\dot{V}$o_2_ of 230 ml min^−1^ minus 20 ml min^−1^ of CO_2_ output into the alveoli).^18^ Airway patency is one prerequisite to allow oxygen to move into the apnoeic lung. Apnoeic oxygenation can be delivered either by standard oxygen flow via an airway catheter, a standard nasal cannula or HFNO (sometimes called Nasal Oxygen During Attempts Securing a Tube—NODeSAT).^18^ High-flow nasal oxygen applied in cases of predicted difficult anatomical airways in the perioperative environment increases safe apnoea time, oxygenation and first-pass success rates.^18^ In critically ill patients undergoing tracheal intubation, apnoeic oxygenation is associated with a lower degree of desaturation during intubation.^13^

The combination of NIV and apnoeic oxygenation was investigated in the proof-of-concept OPTINIV study, which enrolled patients with severe hypoxaemia requiring intubation, and showed that the combined HFNO and NIV technique may be superior to NIV alone in the prevention of hypoxaemia.^19^ Clinicians should be aware of the potential for increased inspiratory and expiratory airway pressures resulting from the combination of HFNO with NIV.^19^

## Intravenous anaesthetic agents and technique

Appropriate selection of i.v. anaesthetic agents is of utmost importance given the haemodynamic effects observed with many agents. The choice of medications for a modern rapid-sequence induction-intubation in the ICU or ED should consider new insights and evidence that challenges the traditional components of the rapid-sequence induction.^3^

Propofol, used in 41.5% of global ICU intubation procedures, is a known modifiable factor independently associated with post-intubation cardiovascular collapse and cardiac arrest.^1^^,^^9^ Ketamine and etomidate have been suggested by recent guidelines as the i.v. anaesthetic agents of choice in critically ill patients, given their favourable haemodynamic profile.^3^^,^^8^^,^^20^

Concerns have been raised over the use of etomidate because of the associated adrenal insufficiency and mortality arising from 11-beta-hydroxylase inhibition; this is described not only after prolonged infusion, but also after a single dose of etomidate in patients with sepsis and septic shock.^21^ More recently, the single-centre EvK trial compared etomidate with ketamine in 791 patients undergoing tracheal intubation in the ICU, and showed a significantly lower 7-day survival after etomidate compared with ketamine (77.3% *vs* 85.1%), although 28-day survival was not significantly different.^22^ This result is congruent with a 2024 Bayesian analysis showing, with moderate probability, that induction with ketamine is associated with a reduced mortality risk compared with etomidate.^23^ Despite the above, we consider that there is no definitive evidence to support ketamine over etomidate and therefore both drugs may be considered for induction of anaesthesia in critical care.^20^

There are several options of device for use during rapid-sequence induction techniques. First, gentle facemask ventilation, applied between induction of anaesthesia and tracheal intubation at 15–17 cmH_2_O, in ICU patients at low risk of pulmonary aspiration, leads to significantly increased oxygen saturations and lower incidence of severe hypoxaemia, compared with a period of no ventilation before intubation.^24^ Second, we recommend clinicians carefully reconsider the risks and benefits of cricoid force application (the Sellick manoeuvre), paying regard to the lack of published benefit in prevention of pulmonary aspiration the known worse laryngeal exposure reported in several clinical trials.^3^^,^^25^ Third, clinicians should be cognisant of the potential role of point-of-care ultrasound for airway evaluation including identification of cricothyroid anatomy and assessment of gastric filling status.^26^ Finally, clinicians should be aware that awake tracheal intubation is not only a perioperative intervention and may be suitable in selected patients in the ICU and ED to maximise first-pass success at tracheal intubation.^1^

## Laryngoscopy and airway devices

Although technological advances lead to new airway devices being brought to market on a regular basis, credible data to support novel devices are often lacking. Where performance data do exist, they are often derived from elective or perioperative settings, which may not be directly and wholly translatable to ICU practice.

We consider videolaryngoscopy (VL) to be one of the most important airway innovations of recent decades. The potential for VL to help the operator overcome difficult airway anatomy and improve first-pass intubation success in the perioperative setting is now well established,^6^^,^^27^, ^28^, ^29^ and we consider VL to hold several advantages applicable to the ICU. First, VL dramatically improves glottic visualisation in situations of reduced cervical spine mobility (e.g. post-traumatic in-line immobilisation), frequently encountered in the ICU. Secondly, using VL reduces the incidence of oesophageal intubation, reported in 5.6% of procedures.^1^ Thirdly, the shared visualisation of laryngeal structures via a video screen, shifts intubation from a single operator duty to a team task. By doing so, using VL allows expert team members to provide focused and prompt support (e.g. external laryngeal manipulation), to overcome challenges or anticipate alternative plans (e.g. adoption of an intubation adjunct or preparation for alternative airway strategies). There is also a non-technical benefit accrued by using VL, in that this shared approach disseminates the emotional burden of emergency tracheal intubation away from the single operator and toward the team, thereby reducing cognitive biases, communication issues and delays in decision-making.^30^

Videolaryngoscopy is not without drawbacks. As with direct laryngoscopy, blood or secretions may compromise visualisation of the glottis, and environmental illumination may reduce screen visibility. These limitations have been, at least in part, overcome by recent technological advances applied to newer devices. In early trials testing VL in critically ill patients, VL did not appear to improve first-pass intubation success and was associated with a higher incidence of physiological adverse events compared with direct laryngoscopy.^31^^,^^32^ The findings may have arisen because the deceptively clear laryngeal view obtained with VL does not always translate to easy intubation of the trachea, leading to prolonged instrumentation and desaturation, especially in the hands of inexperienced operators.^2^ As with any airway management tool, effectiveness of VL in the ICU depends on the operator’s experience and inclusion of VL in daily intubation practice improves operator success.^33^ More recent evidence, including a subgroup analysis of the INTUBE study, demonstrated higher first-pass intubation success with VL compared with direct laryngoscopy, despite a higher prevalence of anatomical predictors of difficult airway management in the VL group.^34^ This analysis also showed no association between VL and adverse events when adjusted for the operator's expertise. Table 1 describes the most relevant findings of recent trials on VL in critical care. UK national guidelines recommend that VL should be available and considered as an option for all intubations of critically ill patients.^8^

**Table 1:** Findings of most relevant studies on videolaryngoscopy use in the critical care setting. DL, direct laryngoscopy; RR, risk ratio.

Year	Authors	Study design	VL type	Main findings
2016	Janz and colleagues^31^	Randomised study	98.6% Macintosh-type	Despite better glottic visualisation with VL, no difference was detected on the primary outcome of first-pass success between VL and DL (adjusted OR for VL 2.02; 95% CI 0.82–5.02, *p*=0.12). No difference was detected on complications, lowest arterial oxygen saturation and in-hospital mortality.
			1.4% Hyperangulated	
2017	Lascarrou and colleagues^32^	Randomised study	Macintosh-type	Videolaryngoscopy did not improve first-pass success (67.7%) compared with DL (70.3%), absolute difference, −2.5% (95% CI −11.9% to 6.9%); *p*=0.60. Videolaryngoscopy was associated with a higher rate of life-threatening complications compared with DL.
2022	Hansel and colleagues^28^	Meta-analysis	Macintosh-type, hyperangulated and channelled	Videolaryngoscopy of any design likely reduces rates of failed intubation (Macintosh-type: RR=0.41; 95% CI 0.26–0.65; hyperangulated: RR=0.51; 95% CI 0.34–0.76).
2023	Russotto and colleagues^34^	Observational study	76% Macintosh-type	Videolaryngoscopy improved first-pass intubation success compared with DL (OR 1.40, 95% CI 1.05–1.87) despite a higher incidence of difficult predictors in the VL group. Videolaryngoscopy was not associated with an increase of adverse events.
			24% Hyperangulated	
2023	Prekker and colleagues^35^	Randomised study	86% Macintosh-type	Videolaryngoscopy improved first-pass intubation success compared with DL (absolute risk difference 14.3%; 95% CI 9.9–18.7; *p*<0.001). No difference was detected on incidence of severe complications nor oesophageal intubation.
			14% Hyperangulated	

It should be noted that although many investigations of VL devices apply a Macintosh-type blade, hyperangulated blades have the theoretical advantage of improving laryngeal exposure. In a recently published trial of 7736 patients undergoing surgery, VL using a hyperangulated blade increased first-pass intubation success compared with direct laryngoscopy (proportional OR for number of intubation attempts 0.20, 95% CI 0.14–0.28; *p*<0.001).^27^ However, further trials are required to elucidate the indications for hyperangulated blade VL in critically ill patients and its potential for shifting the main difficulty from visualisation of the glottis to tracheal tube insertion.^2^ Airway adjuncts (e.g. stylets or tracheal introducers) to facilitate and improve the success rate of intubation with VL are important in this regard. There is conflicting evidence on whether any specific adjunct device is superior when using VL.^36^^,^^37^ Differences observed between studies may arise as much from differential institutional training and expertise, both vital in determining performance of a device.

It is important to emphasise that increased VL availability and proficiency should not justify a reduced availability and training in fibreoptic airway endoscopy. Videolaryngoscopy is not a substitute for fibreoptic endoscopy for airway inspection, endobronchial toilet, microbiological sampling and assistance during other interventions (e.g. percutaneous tracheostomy, confirmation of tracheal tube tip position, selective bronchial intubation).^38^

Finally, there is a limited, but important, role for supraglottic airway devices (SADs) in the ICU.^39^ Despite the design evolutions of second- and third-generation SADs, they remain intrinsically limited in their ability to protect the patient from pulmonary aspiration, provide access to the lower airways and prevent airway device-related trauma arising from prolonged mechanical ventilation. Their fundamental role, as rescue devices in the event of failed tracheal intubation, remains, but clinicians should be aware that in critically ill patients who have already been intubated for a prolonged period, oedema at the laryngeal inlet may impede attempts to oxygenate via SADs sited in an emergency.^40^

## Capnography to confirm successful intubation

Oesophageal intubation occurs in almost 6% of intubations in critical care and if not promptly recognised and acted upon, leads to severe hypoxaemia, cardiac arrest and death.^1^ Waveform capnography is the gold standard method to confirm successful tracheal tube placement.^8^^,^^30^ Lack, or misinterpretation, of capnography was contributory to 74% of the cases of death or persistent neurologic injury reported in the 4th National Audit Project of the Royal College of Anaesthetists.^4^ In the INTUBE observational study reporting critical care intubation practices in 29 countries worldwide, only 25% of intubations were confirmed by waveform capnography and in almost 70% of cases of oesophageal intubation, capnography was being used.^1^ Chest auscultation and other clinical signs perform poorly at confirming tracheal tube placement, especially in critically ill patients.^41^ Waveform capnography should be used whenever an airway procedure or sedation is performed.^8^^,^^30^

## Team working and non-technical skills

The ICU and ED are cognitively complex environments in which to conduct airway management: the quantity of data to be interrogated for safe decision-making often exceeds that which can be held in the conscious working memory.^42^ Background noise and communication issues, ergonomics of spaces, familiarity (or unfamiliarity) of team members with each other and the substantial emotional burden of emergency scenarios should be taken into account.

In such settings, non-technical skills and human factors should be addressed as a specific target of education, training and performance improvement, including through simulation as an assessment of core competencies.^8^ Strategies to be considered include switching from an individual to a teamwork perspective, improving communication skills, promoting the use of cognitive aids (such as visually simple algorithms and checklists), establishing constructive briefing, debriefing and auditing of airway performance as routine clinical practice.^8^ The ergonomics of working environments should always consider the needs imposed by emergency airway management.^8^

## MCQs

The associated MCQs (to support CME/CPD activity) will be accessible at www.bjaed.org/cme/home by subscribers to *BJA Education*.

## Declarations of interests

The authors declare that they have no conflicts of interest.
